# Pediatric cancer patients vaccinated against SARS-CoV-2—a clinical and laboratory follow-up

**DOI:** 10.1007/s00520-024-08422-5

**Published:** 2024-03-11

**Authors:** Benjamin Siebald, Andreas H. Groll, Sarah Salou, Andreas Boldt, Sabine Seiffert, Ulrich Sack, Judith Reemtsma, Christian Jassoy, Jan-Henning Klusmann, Sandra Ciesek, Sebastian Hoehl, Thomas Lehrnbecher

**Affiliations:** 1 Department of Pediatrics, Division of Hematology, Oncology and Hemostaseology, Goethe University Frankfurt, Theodor-Stern-Kai 7, 60590 Frankfurt am Main, Germany; 2 Department of Pediatric Hematology/Oncology, Infectious Disease Research Program, Center for Bone Marrow Transplantation, University Children’s Hospital Muenster, Muenster, Germany; 3 Division of Pediatric Hematology and Oncology, Department of Pediatric and Adolescent Medicine, University Medical Center Freiburg, University of Freiburg, Freiburg, Germany; 4 Medical Faculty, Institute of Clinical Immunology, University of Leipzig, Leipzig, Germany; 5 Institute for Medical Microbiology and Virology, University Hospital and Medical Faculty, University of Leipzig, Leipzig, Germany; 6 Institute of Medical Virology, University Hospital Frankfurt, Johann Wolfgang Goethe University, Frankfurt Am Main, Germany

**Keywords:** Pediatrics, Cancer, Immunocompromised, SARS-CoV-2, Vaccination, Follow-up

## Abstract

**Background:**

Vaccination against SARS-CoV-2 is recommended for cancer patients. However, long-term data on the effectiveness in the pediatric setting are lacking.

**Methods:**

Pediatric patients < 18 years on active treatment for cancer and without prior SARS-CoV-2 infection received three doses of an mRNA vaccine. The clinical course and humoral and cellular immunity were evaluated at the end of the follow-up period of ≥ 1 year after the third dose of vaccine.

**Results:**

SARS-CoV-2 infection occurred in 17 of 19 analyzed patients (median age 16.5 years) during the follow-up period (median 17 months), but no severe symptoms were seen. At ≥ 1 year after the last SARS-CoV-2 antigen exposure, 4 of 17 patients had received the recommended booster vaccine. At the end of the follow-up period, all evaluable 15 patients had anti-SARS-CoV-2 receptor-binding domain IgG antibodies. Twelve of the 15 patients had neutralizing antibody titers ≥ 1:10 against the Delta variant and 12/15 and 13/15 against the BA.1 and BA.5 variants, respectively. Specific T cells against SARS-CoV-2 antigens were seen in 9/13 patients.

**Conclusions:**

Most SARS-CoV-2-vaccinated pediatric cancer patients had SARS-CoV-2 infections and limited interest in booster vaccination. At 1 year after the last antigen exposure, which was mostly an infection, humoral immune responses remained strong.

**Trial registration:**

German Clinical Trials Register DRKS00025254, May 26, 2021.

## Introduction

It has become clear that children and adolescents receiving therapy for cancer or undergoing hematopoietic cell transplantation are at an increased risk for severe or even lethal infection with severe acute respiratory syndrome coronavirus 2 (SARS-CoV-2) [1, 2]. Whereas the rapid development of vaccines against SARS-CoV-2 and the successful implementation of vaccine programs resulted in the decrease in morbidity and mortality in risk groups such as elderly individuals or in immunocompromised adults [3, 4]; there is limited information on the effectiveness of SARS-CoV-2 vaccination in pediatric patients on active cancer treatment, and data on the long-term follow up are lacking [5–7]. We recently reported on the results of a prospective longitudinal study in 21 pediatric patients receiving chemotherapy for cancer which demonstrated that 3 doses of SARS-CoV-2 mRNA vaccine resulted in both humoral and cellular immunity in most patients [6]. Here, we present the follow-up of these patients for at least 1 year after the third dose of the vaccine and evaluate both the clinical course as well as the humoral and cellular immune responses to SARS-CoV-2.

## Patients and methods

Immunocompromised pediatric cancer patients up to 18 years of age, who were on active treatment for any malignancy, were eligible to be enrolled in the study. As previously described in detail [6], exclusion criteria were previous or ongoing infections with SARS-CoV-2, pregnancy, and primary immunodeficiency. Patients were vaccinated with the mRNA vaccine BNT162b2 (Comirnaty, BioNTech/Pfizer), which was administered preferentially at lymphocyte counts ≥ 1000 cells/µl. Two doses of the vaccine were given within 3–6 weeks, followed by a booster vaccination between 4 weeks and 6 months after the second vaccination, which in some cases was delayed due to cancer treatment or complications of therapy. Approximately 2 weeks after the booster vaccine, the immune response was assessed, an interim analysis was performed, and results were reported [6]. The follow-up period started with the immune response assessment after the third dose of vaccine and was scheduled for at least 1 year. The follow-up period ended with the evaluation of the occurrence and severity of SARS-CoV-2 infections and the assessment of a complete blood count; lymphocyte subsets; immunoglobulin G (IgG) level; antibodies against the receptor-binding domain (RBD) and the nucleocapsid antigen of SARS-CoV-2, neutralizing antibodies against the Delta variant and the Omicron variants BA.1 and BA.5; SARS-CoV-2-specific T cells; and antigen-specific memory B cells. The severity of infection with SARS-CoV-2 was classified by the score given by Dong et al. [8]. As previously described in detail, antibodies against the RBD of SARS-CoV-2 and the nucleocapsid antigen were assessed using the Abbott Alinity I platform (Abbott Laboratories, Abbott Park, Illinois) and neutralizing antibodies in an authentic virus-neutralizing assay [9]. SARS-CoV-2-specific T cells were examined by an ELISPOT assay [T-SPOT COVID (Oxford Immunotec)] using two different SARS-CoV-2-specific antigens [10]. The antigen pools contained peptides of the S1 subunit and RBD of the spike protein and peptides of the nucleocapsid protein, respectively [10]. Antigen-specific memory B cells were assessed by ELISPOT using peripheral blood mononuclear cells (PBMCs) of the participants activated for 5 or 6 days with R848 (1 µg/ml) and 96-well Multiscreen-IP filter plates (Millipore, Merck KGaA), which were coated with recombinant SARS-CoV nucleocapsid protein (NP)- maltose-binding fusion protein (MBP) (2 µg/ml), SARS-CoV-2 RBD (1 µg/ml), influenza virus NP-MBP (2 µg/ml) and tetanus toxoid (5 µg/ml, lot 317,490, GSK Vaccines). The total concentration of antibody-secreting B cells was assessed using anti-IgG coated with mouse anti-human IgG mAb (clone MT91/145; Mabtech AB) [11].

Differences between groups (e.g., dose of vaccine) were analyzed using the Wilcoxon rank test for paired samples. Differences between patients with solid tumors and hematological malignancies were assessed with the Mann–Whitney test. A *p*-value (2-tailed) of < 0.05 was considered statistically significant. Analyses were performed using GraphPad Prism software version 5.0.2 (Graph Pad Software, San Diego, California).

The study was approved by the local Ethical Committees Frankfurt (2021–128), Münster (2021–467-b-S), and Freiburg (2021–1382) and was performed in accordance with the Declaration of Helsinki. The study was registered with the German Registry for Clinical Trials (DRKS00025254). All patients and caregivers provided written informed consent.

## Results

The follow-up period after the third dose of the SARS-CoV-2 vaccine was evaluated in a total of 19 pediatric patients [11 female, 8 male; median age at study entry (range) 16.5 years (13.2–17.9)] (Table 1). One of the 21 patients originally enrolled in the study died of the malignancy during the study, and one patient withdrew his consent. The patients suffered from hematological malignancies (*n* = 13) or solid tumors (*n* = 6). The median length (range) of the follow-up period was 17 (12–21) months. At the beginning of the follow-up (e.g., after the third dose of the SARS-CoV-2 vaccine), two patients received intensive chemotherapy, 13 maintenance chemotherapy, and four patients were already off therapy. At the end of the follow-up period (defined by the final clinical and laboratory evaluation), none of the patients received intensive chemotherapy, five patients were on maintenance therapy, and 14 were off therapy (Table 1).

**Table 1:** Patients’ characteristics

Patient #	Sex/age (years)	Underlying malignancy	Start of follow-up*	Therapy at start of follow-up*	SARS-CoV-2 infection/severity score	4th vaccine (second booster)	Therapy at end of follow-up
1	M/17.4	Hematol	11/2021	None	Yes/1 + 2	No	None
2	M/17.9	Hematol	11/2021	MT	Yes/2	No	None
3	F/16.3	Hematol	12/2021	MT	Yes/2	No	None
4	F/17.0	Hematol	12/2021	MT	Yes/2	No	None
5	F/16.0	Solid tumor	12/2021	MT	Yes/2	No	MT
6	M/13.4	Solid tumor	12/2021	None	No	No	None
7	F/16.0	Hematol	11/2021	MT	Yes/2	No	None
8	M/16.4	Hematol	1/2022	MT	Yes/2	No	None
9	F/14.9	Hematol	12/2021	MT	Yes/2	No	None
10	M/14.4	Hematol	1/2022	MT	Yes/2	No	None
11	M/13.2	Hematol	1/2022	MT	Yes/2	Yes	MT
12	M/17.4	Hematol	1/2022	IT	Yes/2	No	MT
13	F/15.6	Hematol	12/2021	IT	Yes/2	No	None
14	F/17.5	Solid tumor	11/2021	None	No	Yes	None
15	M/16.4	Solid tumor	1/2022	None	Yes/2	No	None
16	F/16.8	Hematol	12/2021	MT	Yes/1	No	None
17	F/16.4	Hematol	1/2022	MT	Yes/2	No	None
18	F/15.7	Solid tumor	1/2022	MT	Yes/2	No	MT
19	F/15.5	Solid tumor	2/2022	MT	Yes/2	No	MT

*M* male, *F* female, *Hematol* hematological malignancy, *IT* intensive therapy, *MT* maintenance therapy

*The start of the follow-up period was defined as the day of the immune response assessment after the third dose of the SARS-CoV-2 vaccine (in most patients approximately 2 weeks after vaccination)

### Clinical course during follow-up

Two out of the 19 patients received a fourth dose of the SARS-CoV-2 vaccine (second booster vaccination) (Table 1). While none of the patients had evidence of infection with SARS-CoV-2 up to the third dose, 17 of the 19 patients had at least one SARS-CoV-2 infection during the follow-up period (16 patients with one infection, one patient (#1) with two infections; positive PCR tests in 15 and positive antigen tests in 3 episodes of infection, respectively). Two patients had an asymptomatic infection (score 1), and 16 infections were associated with mild upper respiratory or gastrointestinal symptoms (score 2). No patient experienced moderate, severe, or critical symptoms (scores 3, 4, and 5). Two patients (#12 and #17) received specific immunoglobulins for COVID-19.

### Laboratory evaluation

Four patients withdrew their consent for an additional blood draw at the end of the follow-up period. The blood samples were drawn at a median of 17 months (12–21) after the third dose of the SARS-CoV-2 vaccine.

The median number (range) of lymphocytes (*n* = 11) was 1587/µl (711–2710), of CD4^+^ T cells 542/µl (337–1200; no patient with counts below the normal value of 300/µl), and of CD19^+^ cells 259/µl (14–510; 3 patients with counts below the normal value of 100/µl). The median (range) of total IgG (*n* = 10) was 854 mg/dl (700–1393).

**Fig. 1:**
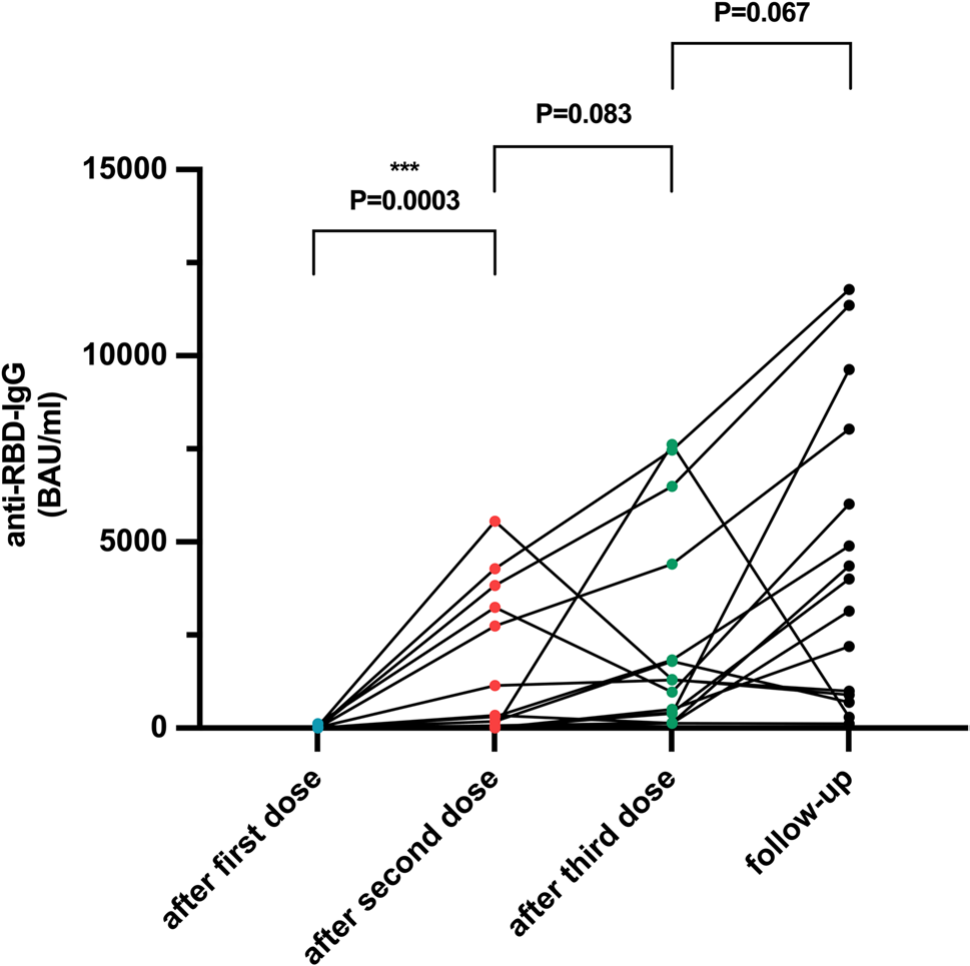
Longitudinal results of the severe acute respiratory syndrome coronavirus 2 (SARS-CoV-2) anti-receptor-binding domain immunoglobulin G (IgG) test. Data points from individual study participants are connected. Differences between groups were assessed using the Wilcoxon matched-pairs signed-rank test. BAU binding antibody units, IgG immunoglobulin G; RBD receptor-binding domain. ***p < .001, the result is statistically significant

**Fig. 2:**
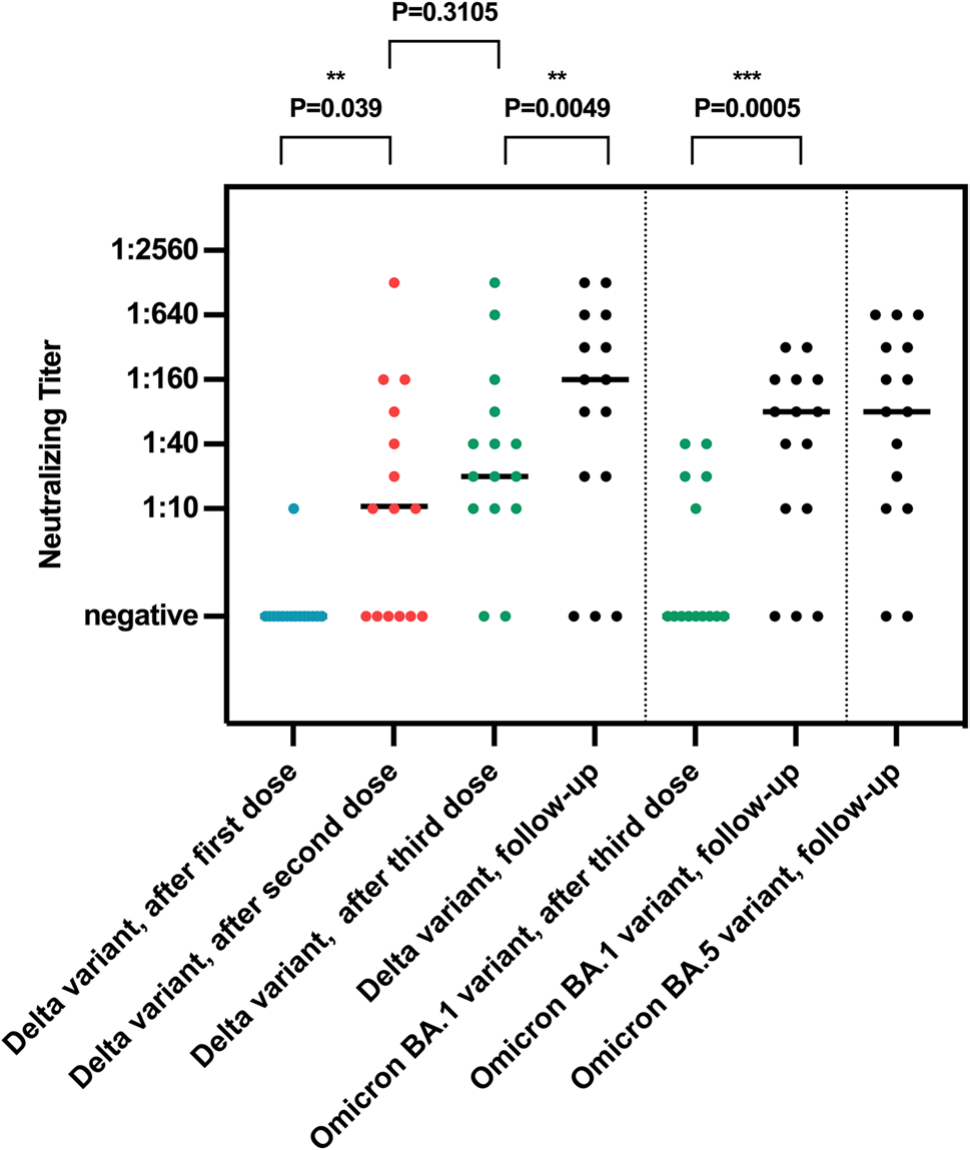
Neutralizing titers against the Delta variant of SARS-CoV-2 after the first, second, and third vaccine doses, as well as at the end of the follow-up period (left), and neutralizing titers against the Omicron variant BA.1 (center) and BA.5 variant (right) after the third dose and/or at the end of the follow-up period. The horizontal line indicates the median titer. Differences between groups were assessed using the Wilcoxon matched-pairs signed-rank test. **p < .01; ***p < .001, results are statistically significant

All 15 patients evaluated had sustained measurable anti-SARS-CoV-2 receptor-binding domain (RBD) IgG antibodies as a marker of immunity (Table 2). Seventeen patients experienced a SARS-CoV-2 infection at a median (range) of 10 (1–15) months prior to the immunological assessment at the end of the follow-up. The geometric mean of anti-SARS-CoV-2 RBD IgG antibodies was 2463.8 BAU/ml, which was higher than after the first (3.8), second (179.9), and third dose of vaccine (1032.3) (Fig. 1). The geometric mean anti-RBD-IgG titer of patients with a hematological malignancy was lower than in those with a solid tumor (2023 and 2305 BAU/ml, respectively), but this difference was not statistically significant (*p* = 0.8581). The two patients without SARS-CoV 2 infection had an RBD IgG titer of 4011.5 BAU/ml (this patient (#14) had received a 4th dose of vaccine) and 296.1 BAU/ml (this patient (#6) had received 3 doses of vaccine), respectively. Seven out of 13 patients (54%) with confirmed infection with SARS-CoV-2 after the third dose of vaccine revealed measurable anti-SARS-CoV-2 nucleocapsid antibodies (Table 2). Compared to the beginning of the follow-up period (approximately two weeks after the third dose of SARS-CoV-2 vaccine), patients had significantly higher mean neutralizing antibody titers against both the Delta variant of SARS-CoV-2 and the BA.1 variant (Fig. 2). Twelve out of 15 evaluated patients had neutralizing antibodies with a titer of at least 1:10 against the Delta and BA.1 variant and 14 out of 15 against the BA.5 variant. The mean titer against both BA.1 and BA.5 variants was 1:80 (Fig. 2).

**Table 2:** Immunologic response against SARS-CoV-2 at the end of the follow-up period

Patient #	Anti-RBD-IgG (BAU/ml)	Anti-NC-IgG (index)	SARS-CoV-2 delta NT	Omikron BA.1-NT	Omikron BA.5-NT	SARS-CoV-2 specific T cells against spike protein	SARS-CoV-2 specific T cells against nucleoprotein
1	2188.20	3.8	1:80	1:40	1:40		
2	997.70	Neg	Neg	Neg	1:10	Pos	Neg
3	4354.70	3.6	1:320	1:80	1:160	Pos	Neg
4	3142.20	Neg	1:80	1:80	1:20	Neg	Neg
5	6024.80	10.8	1:160	1:160	1:320	Pos	Pos
6	295.10	0.5	Neg	Neg	Neg	Neg	Neg
7	898.00	0.8	1:20	1:10	1:10		
8	9637.10	Neg	1:640	1:160	1:320	Pos	Neg
9	11,782.00	4.0	> 1:1280	1:320	1:640	Pos	Neg
10	4594.20	Neg	1:640	1:160	1:640	Pos	Neg
11	695.90	Neg	1:20	1:10	1:80	Pos	Neg
12	118.10	Neg	Neg	Neg	Neg	Neg	Neg
13							
14	4011.50	Neg	1:160	1:40	1:80	Pos	Neg
15							
16							
17							
18	1136.0	3.1	> 1:1280	1:320	1:640	Neg	Neg
19	8034.9	3.0	1:320	1:80	1:160	Pos	Neg

*RBD* receptor-binding domain, *BAU* binding antibody units, *NC* neutralizing assay, *IgG* immunoglobulin G, *NT* neutralizing titer, *Pos* positive, *Neg* negative

At the end of the follow-up period, one out of the 13 patients evaluated showed specific T cells against both the spike and the nucleoprotein, 8 patients showed specific T cells against the spike, but not against the nucleoprotein, whereas in 4 patients, no specific T cells against any of these proteins were detected (Table 2). T-cell responses did not correlate with the humoral response against SARS-CoV-2 (data not shown). Memory B-cell analysis was performed on three of the participants (#5, #9, and #11). At the end of the follow-up period, all of them revealed vigorous memory B cell responses against the SARS CoV-2 RBD and low levels against the SARS CoV-2 nucleoprotein (data not shown).

## Discussion

In a prospective longitudinal study of 21 pediatric patients receiving chemotherapy for cancer, we recently demonstrated that 3 doses of a SARS-CoV-2 vaccine resulted in both humoral and cellular immunity in most of the patients [6]. Here, we present the follow-up of these patients at least 1 year after the third dose of vaccine and evaluate the clinical course, SARS-CoV-2 antibody titers, and specific T cell and memory B cell responses.

The Standing Vaccination Commission (STIKO) of Germany recommends 3 exposures to SARS-CoV-2 antigens (either vaccination or infection) for immunocompromised pediatric and adult patients which should consist of at least two doses of vaccine and an additional booster vaccine after a period of ≥ 12 months [12]. In our study, only two out of the 19 patients received a 4th dose of vaccine. In contrast, 13 out of 17 patients had not received a booster vaccine at 1 year after the last SARS-CoV-2 antigen exposure, which was a SARS-CoV-2 infection in 12 patients and a vaccination in one patient. Although it was speculated that there will be a high rate of acceptance of the SARS-CoV-2 booster vaccination in individuals at increased risk for severe infections [12], data in immunocompromised patients are lacking to date. Our results in immunocompromised pediatric patients reflect the findings of a large population-based panel study performed at the end of 2021 which reported that a considerable proportion of fully vaccinated adults hesitate to receive a booster dose of the SARS-CoV-2 vaccine [13].

At the beginning of 2022, the strict regulations that meant to avoid SARS-CoV-2 infections—such as lockdowns or wearing a face mask—were considerably relaxed in Germany, which most likely accounts for the fact that the vast majority of SARS-CoV-2 infections in our study population occurred during the first 6 months of 2022 with a median (range) of 4 months (0–13) after the third dose of vaccine. Whereas a prospective study of 230 cancer patients who received a third dose of the SARS-CoV-2 vaccine as a booster vaccination reported that in their participants the serological titer cut-off below 803 BAU/ml was predictive of breakthrough infection [14], we found that 9 out of 17 infections occurred in patients with a titer > 803 BAU/ml. In addition, in a study of 2686 adults with varying immune-suppressive diseases who had received two doses of the SARS-CoV-2 vaccine, about 10% of patients had a severe course of the infection or died, and impaired serological and T cell responses were associated with severe infection [15]. In our study population, however, the infections caused mild symptoms at most, which is not surprising, as 90% of our patients had detectable antibody titers after the third dose of vaccine which might have provided protection from severe disease [6].

In 15 patients of our patient population, the serological response to SARS-CoV-2 was assessed after a median (range) of 10 (1–15) months after the last antigen exposure which was a SARS-CoV-2 infection in 13 patients. All patients demonstrated sustained measurable anti-SARS-CoV-2 RBD IgG antibodies as a marker of immunity. In contrast, only 54% of patients with a known infection after the third vaccine dose had measurable anti-SARS-CoV-2 nucleocapsid antibodies, suggesting that this test might not reliably rule out a prior infection.

A total of 12 patients had neutralizing antibodies with a titer of at least 1:10 against the Delta and BA.1 variants of SARS-CoV-2 and 13 against BA.5. Notably, after the BA.1 variant had been the most prevalent variant in Germany during the first half of 2022, the BA.5 variant became the dominant variant (https://www.rki.de/DE/Content/InfAZ/N/Neuartiges_Coronavirus/Virologische_Basisdaten.html;jsessionid=127DD66D7D1E0E47EEEC5E0F07246600.internet052?nn=13490888#doc14716546bodyText5).

Our results are novel, as data on immune responses to SARS-CoV-2 vaccinations in pediatric immunocompromised patients are scarce, in particular, regarding data on long-term follow-up [5–7]. Corroborating our results reported previously [6], one retrospective study reported on good efficacy of SARS-CoV-2 vaccination in 13 patients (median age 17 years) receiving chemotherapy for a solid tumor [7]. Donze et al. [5] evaluated the anti-SARS-CoV-2 immunity in 38 pediatric cancer patients and assessed the humoral, but not the cellular immune response. At 259 days post-vaccination, the probability of maintaining immunity against SARS-CoV-2 declined to 50% in the 15/38 patients who acquired post-vaccination immunity during the first 3 months after vaccination [5]. In comparison, our study shows a sustained humoral response at the end of the follow-up period which can be explained by the fact that we did not remove patients with a documented SARS-CoV-2 infection, as this reflects the real-life setting. In contrast, we observed that 4 out of 13 patients had no detectable anti-SARS-CoV-2 T cells; although, after the second dose of the SARS-CoV-2 vaccine, specific T cells had been demonstrated in two of them [6]. Data in adults suffering from lung cancer showed that the T cell immune response against SARS-CoV-2 at 6 and 12 months after the third dose of vaccine did not significantly differ, but these patients received immunotherapy for their cancer [16].

We recognize that our study population is small, although our analysis included the largest number of reported pediatric cancer patients receiving the recommended three doses of vaccine. In addition, due to a change in SARS-CoV-2 variants, the clinical severity of a SARS-CoV-2 infection has been alleviated considerably since the beginning of 2021, and there is no proof that the course of infection would have been more severe without the vaccination. Notably, our real-life analysis includes the assessment of both humoral and cellular immunity against SARS-CoV-2 and followed the patients for more than 1 year after the third dose of the vaccine. Nevertheless, larger series are mandatory to confirm our data and to monitor the results with emerging variants of SARS-CoV-2 and will be the basis for future recommendations for vaccination against SARS-CoV-2 in immunocompromised patients.

In conclusion, our findings show that despite repeated consultations the majority of pediatric cancer patients have currently limited interest in booster vaccinations against SARS-CoV-2. The results suggest that infections with SARS-CoV-2 are common in these patients and lead to detectable humoral immune response after one year of the last antigen exposure, including neutralizing antibodies against the dominant variant at the time of infection.

## Data Availability

All data and materials are available from the authors on request.
